# *Treponema denticola* enolase contributes to the production of antibodies against ENO1 but not to the progression of periodontitis

**DOI:** 10.1080/21505594.2018.1496775

**Published:** 2018-08-16

**Authors:** Ahreum Lee, Yong C. Kim, Keumjin Baek, Jehan Alam, Yun S. Choi, Yaeeun Rheu, Yoo Jin Shin, Sungtae Kim, Hyun-Duck Kim, Yeong W. Song, Youngnim Choi

**Affiliations:** a Department of Immunology and Molecular Microbiology, School of Dentistry and Dental Research Institute, Seoul National University, Seoul, Korea; b Department of Periodontology, School of Dentistry and Dental Research Institute, Seoul National University, Seoul, Korea; c Department of Preventive and Social Dentistry, School of Dentistry, Seoul National University; d Department of Internal Medicine, Seoul National University Hospital; e Department of Molecular Medicine and Biopharmaceutical Sciences, Graduate School of Convergence Science and Technology and College of Medicine, Seoul National University, Seoul, Korea

**Keywords:** *Treponema denticola*, enolase, periodontitis, autoantibody, TNFα

## Abstract

Autoantibodies against alpha-enolase (ENO1) are often detected in various infectious and autoimmune diseases. Anti-ENO1 antibody titers were reported to be associated with the severity of periodontitis in patients with rheumatoid arthritis. Because the enolase of the periodontal pathogen *Treponema denticola* (TdEno) has the highest homology with ENO1 among the enolases of human-associated bacteria, we hypothesized that anti-ENO1 autoantibodies produced during the immune response to TdEno may contribute to the progression of periodontitis and tested it in human and mouse systems. In human subjects with healthy periodontium or chronic periodontitis, a strong positive correlation between the levels of anti-TdEno and anti-ENO1 antibodies was observed. In addition, the purified anti-TdEno antibodies recognized ENO1 as well as TdEno in a dot blot, confirming the cross-reactivity between TdEno and ENO1. However, anti-ENO1 antibody titers were not associated with the severity of periodontitis. To further investigate the role of TdEno in the production of anti-ENO1 antibodies and the progression of periodontitis, mice received an oral gavage of *P. gingivalis* alone, subcutaneous immunization with TdEno alone, or both *P. gingivalis* oral gavage and TdEno immunization. Immunization with TdEno induced not only anti-TdEno but also anti-mouse Eno1 (mEno1) antibodies and increased the expression of TNFα in the gingival tissues. However, alveolar bone loss was not increased by TdEno immunization. In conclusion, autoreactive anti-ENO1/mEno1 antibodies that are produced as byproducts during the antibody response to TdEno play a minimal role in the progression of periodontitis in the absence of rheumatoid arthritis.

## Introduction

Periodontitis is caused by dysbiosis of the subgingival biofilm that accompanies increases in the amounts of periodontal pathogens such as *Porphyromonas gingivalis, Treponema denticola*, and *Tannerella forsythia* [1]. Accumulating evidence supports an association between periodontitis and rheumatoid arthritis (RA). RA patients have more tooth loss and a high incidence of periodontitis compared with non-RA subjects [2–6]. Conversely, patients with periodontitis are more likely to suffer from RA than periodontally healthy individuals [7,8]. Such epidemiological associations might be attributed to shared common risk factors of periodontitis and RA, such as HLA-DRB1 alleles and smoking [9]. However, it has been shown that the association with periodontitis is independent of all covariates in anti-citrullinated protein antibody-positive RA [10]. The presence of anti-citrullinated protein antibodies is a specific marker for RA, and several citrullinated proteins, including fibrinogen, vimentin, collagen type II, and human enolase 1 (ENO1, also called α-enolase), serve as autoantigens [11]. *P. gingivalis* has been suggested as a link in the association between RA and periodontitis: *P. gingivalis* expresses peptidylarginine deiminase, which can produce citrullinated peptides upon incubation with human fibrinogen or ENO1 [12]. Furthermore, *P. gingivalis* enolase is cross-reactive with the immunodominant epitope of citrullinated ENO1 in RA patients. [13]

The affinity-purified antibodies to the citrullinated epitope of ENO1 also react with native ENO1 [13]. Antibodies to ENO1 have been reported in a variety of infectious and autoimmune diseases, including RA [14]. Although ENO1 is a glycolytic enzyme ubiquitously expressed in the cytosol, it is also expressed on the surface of stimulated leukocytes, such as neutrophils, lymphocytes, and monocytes [15]. Inflammatory stimuli induce rapid translocation of ENO1 from the cytosol to the cell surface, and cell-surface ENO1 serves as a receptor and activator of plasminogen, which assists inflammatory cell infiltration [15]. In addition, stimulation of cell surface ENO1 with anti-ENO1 antibodies induces the robust production of inflammatory cytokines from mononuclear leukocytes [16]. A recent study showed that anti-ENO1 antibody titers are associated with the severity of periodontitis as well as RA disease activity in RA patients [17].

Features of autoimmunity have been reported in periodontitis. Anti-type I collagen antibody-producing cells and type I collagen-specific T cell clones have been identified in inflamed gingival tissues [18,19]. Periodontitis patients have elevated levels of serum antibodies to human heat shock protein 60 and antibodies to *P. gingivalis* GroEL that are cross-reactive to each other [20]. ENO1 is a highly conserved protein from prokaryotes to eukaryotes, with 40–90% identity between enolases from different species [21]. A similarity search of the bacterial protein database for ENO1 revealed that the enolase of *T. denticola* (TdEno) has the highest score among those of human-associated bacteria [22]. Therefore, we hypothesized that anti-ENO1 antibodies produced by molecular mimicry during the antibody response to TdEno may contribute to the progression of periodontitis. The aim of this study was to investigate the role of TdEno in the production of anti-ENO1 antibodies and the progression of periodontitis.

## Results

### The cross-reactivity of anti-TdEno antibodies with anti-ENO1 in human subjects

**Figure 1.**
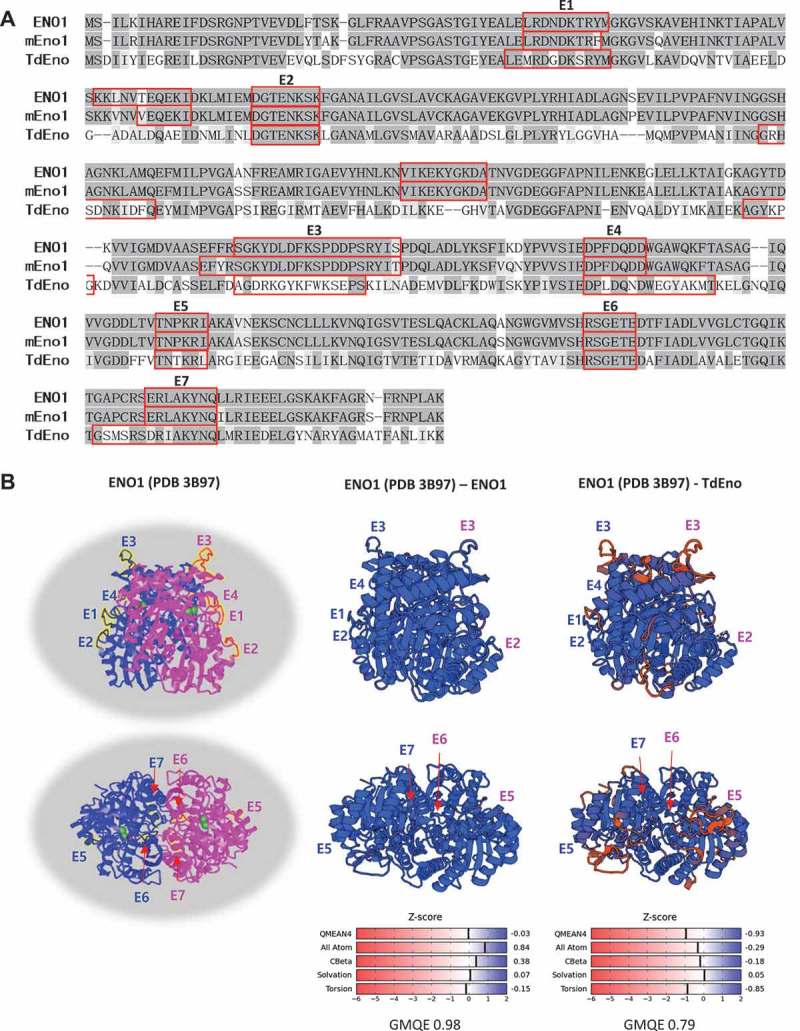
The homology of TdEno with ENO1 and mEno1. (a) The amino acid sequences of ENO1, mEno1, and TdEno were aligned. Compared with ENO1, identical and conserved amino acids are highlighted in dark and light grey, respectively. B cell epitopes predicted using the Emini Surface Accessibility Prediction method in each protein were marked with red squares. The epitopes of ENO1 and TdEno located at the overlapping regions of aligned sequences are labeled from E1 to E7. (b) The seven epitopes are highlighted in yellow at the known structure (PDB 3B97) of ENO1 (left panel). To confirm the reliability of structure homology modeling, the structure of ENO1 was constructed at the SWISS-MODEL server using the PDB 3B97 as a template (middle panel). The structure homology of TdEno with ENO1 is shown in the right panel.

TdEno had 54% identity and 70% homology with ENO1 and 53% identity and 70% homology with mouse Eno1 (mEno1). In addition, seven (E1 – E7) out of nine predicted B cell epitopes from each protein were located at the overlapping regions of the aligned sequences (Figure 1(a)). Because TdEno is just one amino acid shorter than ENO1 and mEno1, TdEno is expected to have a similar three-dimensional structure. The modeling of TdEno structure on the known ENO1 structure using the SWISS-MODEL server resulted in 0.79 and −0.93 for Global Model Quality Estimation (GMQE) and QMEAN4 scores, respectively, which reflected quite high reliability and the high quality of prediction. The predicted three-dimensional structure of TdEno was very close to that of ENO1. Furthermore, six predicted B cell epitopes of TdEno were located at the structurally conserved regions (Figure 1(b)).

**Figure 2.**
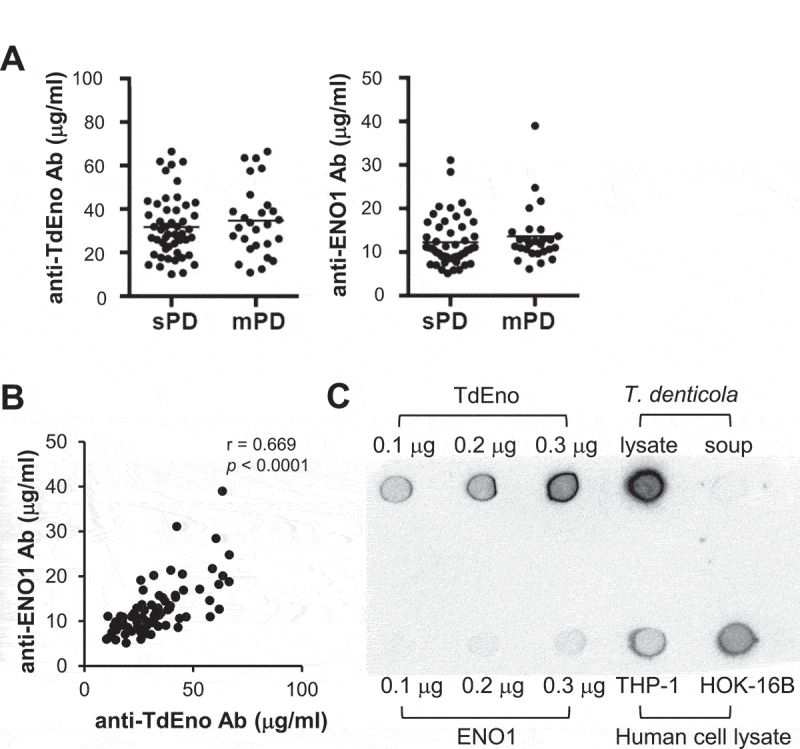
The cross-reactivity of anti-TdEno antibodies with ENO1 in human subjects (a) Concentrations of anti-TdEno and anti-ENO1 IgG antibodies in sera from subjects with slight (sPD) or moderate (mPD) chronic periodontitis were determined by ELISA. (b) Spearman’s correlation between the levels of anti-TdEno and anti-ENO1 antibodies is shown. (c) Various amounts of recombinant TdEno and ENO1 proteins, the lysate and culture supernatant of *T. denticola*, and the lysates of human cell lines (THP-1 and HOK-16B) were subjected to dot blotting using anti-TdEno antibodies that were affinity-purified from the pooled sera.

To investigate the role of TdEno in the production of anti-ENO1 antibodies and the progression of periodontitis, sera from subjects with slight (n = 50) or moderate (n = 27) chronic periodontitis were obtained. The group with moderate periodontitis presented higher age, plaque index (PI), gingival index (GI), bleeding on probing (BOP), probing pocket depth (PPD), and clinical attachment level (CAL) than the slight group (Table 1). When the concentrations of IgG antibodies to TdEno and ENO1 were determined by ELISA, there was no significant difference between groups (Figure 2(a)). When the effect of anti-ENO1 antibody titers on the severity of periodontitis was assessed with the adjustments of important risk factors for periodontitis, such as age, smoking, and PI, a significance was not observed (Odd Ratio: 1.95, 95% Confidence Interval 0.08 – 45.58, *p* = 0.679). To determine the levels of anti-TdEno and anti-ENO1 antibodies in periodontally healthy subjects, sera from healthy subjects (n = 20) and patients with slight periodontitis (n = 23) were additionally obtained (Supplementary Table 1). Although the differences were not significant, both the anti-TdEno and anti-ENO1 antibody titers tended to be lower in healthy subjects than in patients with slight periodontitis (Supplementary Figure 1A). The anti-ENO1 antibody titers did not have a significant association with the development of slight periodontitis, either (Odd Ratio: 43.2, *p* = 0.333).

**Table 1.** Periodontal parameters of non-RA human subjects with slight or moderate periodontitis.

	Slight periodontitis (n = 50)	Moderate periodontitis (n = 27)	*p* value
Age*	56.9 ± 13.3	62.0 ± 9.0	0.047^†^
Female %	88	85	0.734^ǂ^
Smoking %	4	3.7	0.720^ǂ^
The number of tooth*	26.0 ± 2.8	25.3 ± 2.9	0.401
Plaque index (PI) *	0.58 ± 0.25	0.87 ± 0.36	< 0.0001^†^
Gingival index (GI) *	0.10 ± 0.15	0.20 ± 0.18	0.027^#^
Bleeding on probing (BOP) *	9.6 ± 7.2	15.3 ± 11.9	0.035^#^
Probing pocket depth (PPD) *	1.65 ± 0.13	1.92 ± 0.28	< 0.0001^†^
Clinical attachment level (CAL) *	2.59 ± 0.21	3.47 ± 0.45	< 0.0001^#^

*mean ± standard deviation, ^ǂ^by Fisher’s exact test, ^†^by t-test, ^#^by Mann-Whitney U test

Spearman’s rho revealed a strong positive correlation between the levels of anti-TdEno and anti-ENO1 antibodies, suggesting potential cross-reactivity (Figure 2(b) and Supplementary Figure 1B). To confirm the cross-reactivity between TdEno and ENO1, dot blotting was performed using TdEno-specific antibodies that were purified from pooled sera. The TdEno-specific antibodies reacted with ENO1 and the lysates of human cell lines HOK-16B and THP-1 as well as TdEno and the lysates and culture supernatant of *T. denticola* (Figure 2(c)).

### Immunization with TdEno induced the production of anti-mEno1 antibodies but not the progression of periodontitis in mice

**Figure 3.**
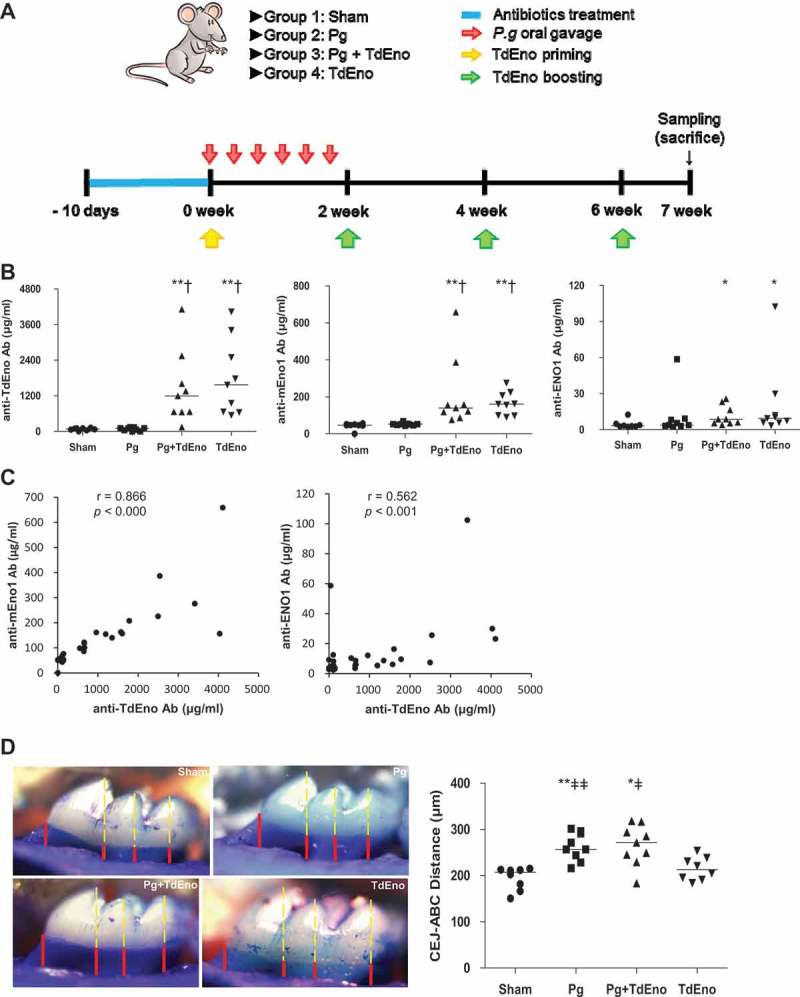
The effect of TdEno-induced anti-mEno1 antibodies on the progression of periodontitis in mice (a) The experimental scheme is shown. (b) Concentrations of anti-TdEno, anti-mEno1, and anti-ENO1 IgG antibodies in mouse sera were determined by ELISA. (c) Spearman’s correlation between the levels of anti-TdEno and either anti-mEno1 or anti-ENO1 antibodies is shown. (d) Images of the maxillary first molar of a representative mouse from each group are shown. Distances between the cemento-enamel junction (CEJ) and alveolar bone crest (ABC) at four lingual sites per the first molar were measured (left panel). The mean value for each mouse was graphed (right panel). The horizontal line presents the mean of each group. *p < 0.05 and **p < 0.005, compared to sham, †*p* < 0.0001, compared to Pg, and ǂ*p* < 0.05 and ǂǂ*p* < 0.05, compared to TdEno.

To further investigate the role of TdEno in the production of anti-ENO1 antibodies and the progression of periodontitis, mice that received oral gavage of *P. gingivalis* alone (Pg), subcutaneous immunization with TdEno alone (TdEno), or both *P. gingivalis* oral gavage and TdEno immunization (Pg+ TdEno) were compared with the sham group (Figure 3(a)). Immunization with TdEno induced not only anti-TdEno antibodies but also anti-mEno1 and anti-ENO1 antibodies (Figure 3(b)). Furthermore, the levels of anti-mEno1 and anti-ENO1 antibodies presented strong positive correlations with those of anti-TdEno (Figure 3(c)). However, immunization with TdEno did not significantly enhance alveolar bone destruction compared with the sham or Pg groups (Figure 3(d)).

### Immunization with TdEno increased the expression of TNFα in the gingival tissues

**Figure 4.**
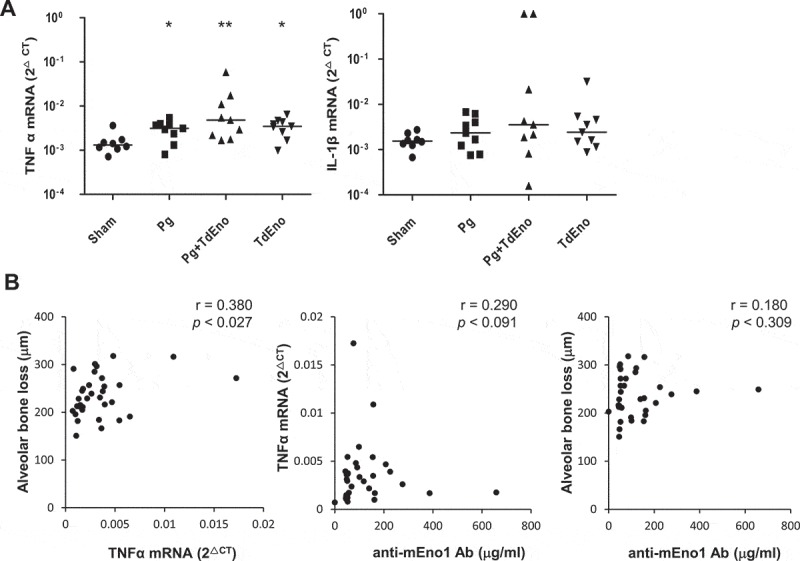
Expression of inflammatory cytokines in the gingival tissues (a) Expression levels of TNFα and IL-1β mRNA in the gingival tissues were measured by real-time PCR. *p < 0.05 and **p < 0.005, compared to sham. (b) Spearman’s correlations between TNFα and alveolar bone loss, anti-mEno1 antibodies and TNFα or alveolar bone loss are shown.

To better understand the role of TdEno in the progression of periodontitis, the expression of the inflammatory cytokines TNFα and IL-1β in gingival tissues was examined by real-time PCR. TNFα expression was significantly increased in all three experimental groups compared with the sham group. Although the Pg+ TdEno group showed a higher median value for the TNFα expression than the Pg group did, the difference was not significant (Figure 4(a)). The expression patterns of IL-1β were very similar to those of TNFα, but none of the inter-group differences were statistically significant (Figure 4(a)). The levels of TNFα in serum were also examined by ELISA, but TNFα was detected in only two mice, one from the Pg group and the other from the Pg+ TdEno group.

The levels of TNFα expression in the gingival tissues had a moderate positive correlation with the degree of alveolar bone loss; however, the levels of anti-mENO1 antibodies did not correlate with either the levels of TNFα expression or alveolar bone loss (Figure 4(b)).

## Discussion

In this study, we present our findings that TdEno, the enolase of the periodontal pathogen *T. denticola*, induces the production of anti-ENO1/mEno1 antibodies through molecular mimicry, but the anti-ENO1/mEno1 antibodies play a minimal role in the progression of periodontitis.

Cross-reactivity between *T. denticola* and host enolases was confirmed by i) a strong positive correlation between the levels of anti-TdEno and anti-ENO1 antibodies in human subjects, ii) a strong positive correlation between the levels of anti-TdEno and anti-mEno1 in mice, and iii) recognition of ENO1 by affinity-purified anti-TdEno antibodies in a dose-dependent manner in a dot blot. In our preliminary animal experiment, the levels of both anti-TdEno and anti-mEno1 antibodies started to fall 4 weeks after the last boost. This suggests that the anti-mEno1 antibodies observed in our system are the byproduct of anti-TdEno antibody production. The cross-reactivity between bacterial enolase and ENO1 was first reported in association with acute rheumatic fever after Group A streptococcal infection [23]. The enolase of *Streptococcus pyogenes* M1 has 46.6% identity with ENO1, and polyclonal antibodies raised against the streptococcal enolase recognized not only recombinant ENO1 but also ENO1 expressed on the surface of leukocytes. Patients with acute rheumatic fever had higher levels of serum antibodies that react with ENO1 and bacterial enolase than did patients with streptococcal pharyngitis or healthy control subjects, suggesting the role of streptococcal enolase as a cross-reactive antigen in post-streptococcal autoimmune diseases [23]. The gingival tissues of periodontal lesions are infected with complex bacterial communities, including *T. denticola* and *P. gingivalis*, and patients with periodontitis frequently experience bacteremia after tooth brushing or even chewing [24,25]. Although TdEno has the highest homology with ENO1 among the enolases of human-associated bacteria, it is expected that not only TdEno but also the enolases of diverse periodontitis-associated bacteria contribute to the production of anti-ENO1 antibodies in periodontitis.

In our murine model of periodontitis, the TdEno group expressed increased levels of TNFα in the gingival tissues compared with the sham group, suggesting the contribution of anti-mEno1 antibodies to TNFα expression. However, no change in the levels of serum TNFα indicates that the induction of TNFα by the anti-mEno1 antibodies must be limited to local tissues where stimulated leukocytes expressing Eno1 on the cell surface are present. Meanwhile, comparable levels of TNFα in all three experimental groups suggest that bacteria as well as the anti-mEno1 antibodies play an important role in TNFα expression in gingival tissues. Consequently, the anti-mEno1 antibody titers did not have a significant correlation with TNFα expression in the gingival tissues.

In periodontitis, the production of anti-TdEno antibodies (or antibodies against other bacterial enolases) is expected to have both destructive and protective roles. Gingival tissues with chronic periodontitis are heavily infiltrated with activated T cells, B cells, monocytes, and neutrophils [26]. The anti-ENO1/mEno1 antibodies produced by molecular mimicry would contribute to tissue destruction through the induction of TNFα from activated leukocytes and immune complex-mediated tissue injury [15,16]. Meanwhile, as shown in *T. forsythia* and *Streptococcus pneumoniae*, bacterial enolases have pathogenic potential because they facilitate bacterial invasion and induce inflammatory cytokine production in monocytes [27,28]. Considering that the enolases are highly conserved, antibodies against any bacterial enolase, including anti-TdEno antibodies, are expected to cross-react with the enolases of other bacterial species [27]. Thus, anti-TdEno antibodies may play a protective role by neutralizing the pathogenic potential of bacterial enolases. This would explain why there is no increase in alveolar bone loss by immunization with TdEno. In our human study, relatively small number of healthy subject samples were used, and a significant association of anti-ENO1 antibody titers with periodontitis may be achieved in a larger cohort. However, a cross-sectional study cannot conclude if the production of anti-ENO1 antibodies is the result of gingival tissue infection with bacteria i.e. periodontitis or a contributing factor to disease progression. In the current study, high levels of anti-mEno1 antibodies were induced in mice by injecting recombinant TdEno at the tail base. Thus, our animal study clearly tells that anti-mEno1 antibodies produced independent of gingival tissue infection do not contribute to periodontal destruction.

Anti-ENO1 antibodies may contribute to the progression of periodontitis in specific conditions, such as RA [17]. In RA patients, a substantial percentage of peripheral blood mononuclear cells and synovial fluid mononuclear cells express ENO1 on the surface [16]. Interaction between anti-ENO1 antibodies and ENO1-positive cells in peripheral blood may contribute to the elevated TNFα serum levels in RA patients [29,30], and the circulating TNFα may in turn contribute to periodontal destruction. When a subset of RA patients with slight periodontitis were randomly selected from the original study population, the RA patients presented higher levels of inflammation indexes GI and BOP, despite the lower levels of PI, PPD, and CAL and compatible levels of anti-TdEno and anti-ENO1 antibodies compared to non-RA subjects (Supplementary Table 2 and Supplementary Figure 2). Notably, the GI and BOP indexes of RA patients with slight periodontitis were as high as those of non-RA subjects with moderate periodontitis presented in Table 1, suggesting a strong inflammatory response in RA. If such strong inflammation leads to faster periodontal destruction in RA needs to be answered in a longitudinal study. In the current animal study, the role of anti-mEno1 antibodies in the progression of periodontitis was evaluated in the absence of RA. A separate study is required to investigate the role of anti-mEno1 antibodies in the progression of periodontitis in a RA model.

Interestingly, immunization of mice with TdEno induced more than ten times higher levels of anti-mEno1 antibodies compared with anti-ENO1 antibodies (*p* < 0.0001), despite having the same degree of homology. During the germinal center reaction, binding to antigens and signaling through the B cell receptors is essential for the selection of the clone and antibody production [31], which may be essential even for autoantibodies. During the antibody response to bacterial antigens in the germinal center, what determines the selection or deselection for B cell clones cross-reactive to autoantigens awaits further clarification.

In conclusion, the antibody response to TdEno results in the production of autoreactive anti-ENO1/mEno1 antibodies that contribute to the expression of TNFα in gingival tissues. However, the role of anti-mEno1 antibodies in the progression of periodontitis is minimal in the absence of RA.

## Materials and methods

### Human sera

This study was approved by the Institutional Review Boards at Seoul National University Hospital (H-1103–151-357) and Seoul National University Dental Hospital (CRI-17,009). In the current study, two sets of human serum samples were used. The first set, including 77 non-RA control cases and 38 RA cases, was chosen from the previous study pool that included 248 RA patients and 85 age- and sex-matched non-RA controls [17]. All subjects had chronic periodontitis, slight or moderate, according to the 1999 criteria of the American Academy of Periodontology [32]. To include periodontally healthy subjects, additional samples from healthy subjects (n = 20) and slight periodontitis patients (n = 23) were obtained through the Department of Periodontology Seoul National University Dental Hospital. Because ELISA analyses for the two sets of sera were performed independently, and the sources of recombinant ENO1 used were different, the two sets of data were presented separately without pooling.

### Blast search and sequence alignment

The human ENO1 protein sequence was subjected to homology search with bacterial proteins in the NCBI database using a blastp program with default settings (word size: 3; matrix: BLOSUM62; Gap costs for existence and extension: 11 and 1, respectively). The sequence of the retrieved enolase of *T. denticola* ATCC 35405 (TdEno, sequence ID: AAS11440) was aligned to those of ENO1 and mEno1 using a GeneDoc software version 2.6.001 (www.psc.edu/biomed/genedoc).

### B cell epitope analysis and structure homology modeling

The amino acid sequences of ENO1, mEno1, and TdEno were subjected to prediction analysis of B cell epitopes using the Emini Surface Accessibility Prediction method provided by the Immune Epitope Database and Analysis Resource (http://tools.immuneepitope.org/main/bcell/). The structure homology of TdEno with ENO1 (structure ID: PDB 3B97) was constructed at the SWISS-MODEL server (https://swissmodel.expasy.org/).

### Recombinant enolases: ENo1, mEno1, and TdEno

Recombinant ENO1 and mEno1 expressed in yeast were purchased from MyBiosource (San Diego, CA, USA). Recombinant ENO1 expressed in Escherichia coli purchased from Novus Biologicals (Littleton, CO, USA) was also used in some experiments. Recombinant TdEno was prepared in house. A 1.3 kb gene fragment encoding TdEno was amplified from the genomic DNA of *T. denticola* ATCC 35405 using a pair of primers, 5ʹ-GGATCCTCTGATATTATTTATATTG-3ʹ and 5ʹ-TTATTTTTTTATCAGGTTTGC-3ʹ, and the Takara Ex Taq^TM^ PCR kit (Takara, Shiga, Japan). The PCR product was first cloned into a TA vector (iNtRON Biotechnology, Seongnam, Gyenggi-do, Korea) and then into the pQE-30 expression vector (QIAGEN, Hilden, Germany) using *Bam* H1 and *Kpn* I sites. The cloned gene was expressed in *Escherichia coli* strain SG 13009 by induction with 1 mM isopropyl β-D-1-thiogalactopyranoside for 4 hours. The recombinant protein containing a His tag was purified using a Ni-NTA slurry (QIAGEN) from clear cell lysates and then refolded by dialyzing against refolding buffer. The prepared TdEno with His tag was 52 kD in an SDS-page gel. Endotoxin was removed from the recombinant protein by Triton X-114 extraction to prevent the capture of LPS-binding antibodies in sera during ELISA. The levels of endotoxin in all recombinant enolases determined by the LAL test were < 0.1 EU per 1 μg of the protein.

### ELISA

To determine the levels of antibodies against enolases in sera, high binding 96-well plates (Costar, New York, NY, USA) were coated with 0.1 μg/well of TdEno, mEno1, or ENO1 in PBS overnight at 4 °C. After blocking with 1% bovine serum albumin in PBS, the plates were incubated with sera at various dilutions. After washing, the plates were incubated with HRP-conjugated goat anti-human IgG (Southern Biotech, Birmingham, AL, USA) or HRP-conjugated goat anti-mouse IgG (Santa Cruz Biotechnology, Dallas, TX, USA). The bound detection antibody was developed with 3, 3ʹ, 5, 5ʹ-tetramethylbenzidine substrate (Sigma-Aldrich, St. Louis, MO, USA). The reaction was stopped by the addition of 2 N H_2_SO_4_, and absorbance was immediately measured at 450 nm. For the standard, two columns in each plate were coated with serially diluted (50 ng/ml to 1.56 ng/ml) human IgG1 (Southern Biotech) or mouse IgG1 (BD, Franklin Lakes, NJ, USA) instead of the antigen. The levels of specific IgG were calculated using an equation generated from the standard curve. To make the optical densities fall within the standard curve, human sera were diluted in blocking buffer at 1:1000 and 1:3000. Mice sera were diluted 1:128000 and 1:64000 for the anti-TdEno antibody ELISA, 1:64000 and 1:32000 for anti-mEno1 antibody ELISA, and 1:1000 and 1:2000 for anti-ENO1 ELISA.

The concentrations of TNFα in the mouse sera were measured using a kit (R&D Systems, Minneapolis, MN, USA) according to the manufacturer’s instructions.

### Dot blot using purified TdEno-specific antibodies

The antibodies specific to TdEno were affinity-purified by incubating pooled human sera with TdEno immobilized onto a nitrocellulose membrane (Merck Millipore, Seoul, Korea). TdEno, *T. denticola* lysate, culture supernatant of *T. denticola*, ENO1, and protein lysates of HOK-16B and THP-1 cells were immobilized onto the nitrocellulose membrane. After blocking with 5% skim milk in 1% TBST, the membrane was then subjected to immunoblotting with the purified anti-TdEno antibodies.

### Preparation of bacteria

*P. gingivalis* ATCC 49417 (American Type Culture Collection, Manassas, VA, USA) was cultured in Brain Hear Infusion broth medium (BD), supplemented with 10 μg/ml vitamin K (Sigma) and 5 μg/ml hemin (Sigma) at 37°C under anaerobic condition (CO_2_ 10%, H_2_ 10%, N_2_ 80%). For oral gavage, 2 × 10^9^ cells of *P. gingivalis* were mixed with 2% carboxymethyl cellulose (TCI, Tokyo, Japan) in 100 μl PBS.

### Experimental periodontitis in mice

Experimental protocols and animal handling procedures were approved by the Seoul National University Animal Care and Use Committee, Seoul, Republic of Korea (No. SNU-150224–1). Specific pathogen-free 6-week-old female BALB/c mice were divided into Sham, Pg, Pg+ TdEno, and TdEno groups. The Pg group (n = 9) received oral gavage of *P. gingivalis* six times at 2-day intervals, the TdEno group (n = 9) received subcutaneous immunization with TdEno four times at 2-week intervals, and the Pg+ TdEno group (n = 9) received both *P. gingivalis* and TdEno immunization. The sham group (n = 8) received only carboxymethyl cellulose and adjuvants. For immunization with TdEno, 4 μg TdEno in 50 μl volume mixed with an equal volume of complete Freund’s adjuvant (Sigma-Aldrich) was subcutaneously injected at the tail base for priming, and 1 μg TdEno in 50 μl volume mixed with an equal volume of incomplete Freund’s adjuvant was used for the boosting. The amount of TdEno and immunization times to induce anti-mEno1 antibodies were determined by preliminary experiments.

Mice were euthanized 7 weeks after the first oral gavage or immunization. From the dissected maxillae, soft tissues around the teeth were first removed for RNA extraction. The left-over bone and teeth were completely defleshed, immersed overnight in 3% hydrogen peroxide, and stained with 1% methylene blue. Lingual images of the maxillary left and right first molar were photographed under a microscope (x40). After coding the images, the distances from the cemento-enamel junction to the alveolar bone crest at the four lingual sites were blindly measured using a microscope with SPOT Advanced software (SPOT Imaging, SterlingHeights, MI, USA).

### Real-time PCR for inflammatory cytokines

Total RNA was extracted from the maxillary gingival soft tissues of mice using easy-BLUE (iNtRON Biotechnology), and then synthesized to cDNA by reverse transcription. The expression levels of TNFα and IL-1β were determined by real-time PCR using SYBR® Premix Ex Taq (Takara). A pair of primers was designed to amplify at least two exons for TNFα: 5ʹ-CCCACGTCGTAGCAAACC-3ʹ and 5ʹ- ACAAGGTACAACCCATCGGC-3ʹ; IL-1β: 5ʹ-TGCCACCTTTTGACAGTGATG-3ʹ and 5ʹ- AAGGTCCACGGGAAAGACAC-3ʹ; and β-actin: 5ʹ-CTGTCGAGTCGCGTCCAC-3ʹ and 5ʹ- TTCCCACCATCACACCCT-3ʹ. Amplification was performed in a fluorescence thermocycler (Applied Biosystems, Foster City, CA, USA) under the following conditions: initial denaturation at 94°C for 4 min, followed by 40 cycles of denaturation at 94°C for 15 sec, annealing at 60°C for 15 sec, and elongation at 72°C for 33 sec. Relative copy numbers compared to β-actin were calculated using 2^−∆Ct^.

### Statistical analysis

To determine differences between two independent groups, the chi-square and Mann-Whitey U tests were used for categorical and continuous variables, respectively. Differences among four independent groups were determined by the Kruskal-Wallis H test followed by the Mann-Whitey U test. Correlations between two variables were determined using Spearman’s correlation coefficients. The association of log transformed anti-ENO1 antibody titers with the severity of periodontitis was determined by multivariate logistic regression analysis. All statistics were performed using SPSS 23.0 (IBM, Armonk, NY, USA). Data were considered statistically significant at a *p*-value of < 0.05.

## Abbreviations

RA: rheumatoid arthritis
ENO1: human enolase 1
mEno1: mouse enolase 1
TdEno: *Treponema denticola* enolase
PI: plaque index
GI: gingival index
BOP: bleeding on probing
PPD: probing pocket depth
CAL: clinical attachment level
