# Supplementary material for: Treponema denticola enolase contributes to the production of antibodies against ENO1 but not to the progression of periodontitis
Source: Virulence. 2018 Aug 16;9(1):1263–72. doi: 10.1080/21505594.2018.1496775 (PMC6104692; doi:10.1080/21505594.2018.1496775)
Supplement: Supplemental Material [file kvir-09-01-1496775-s001.pptx]

## Slide 1
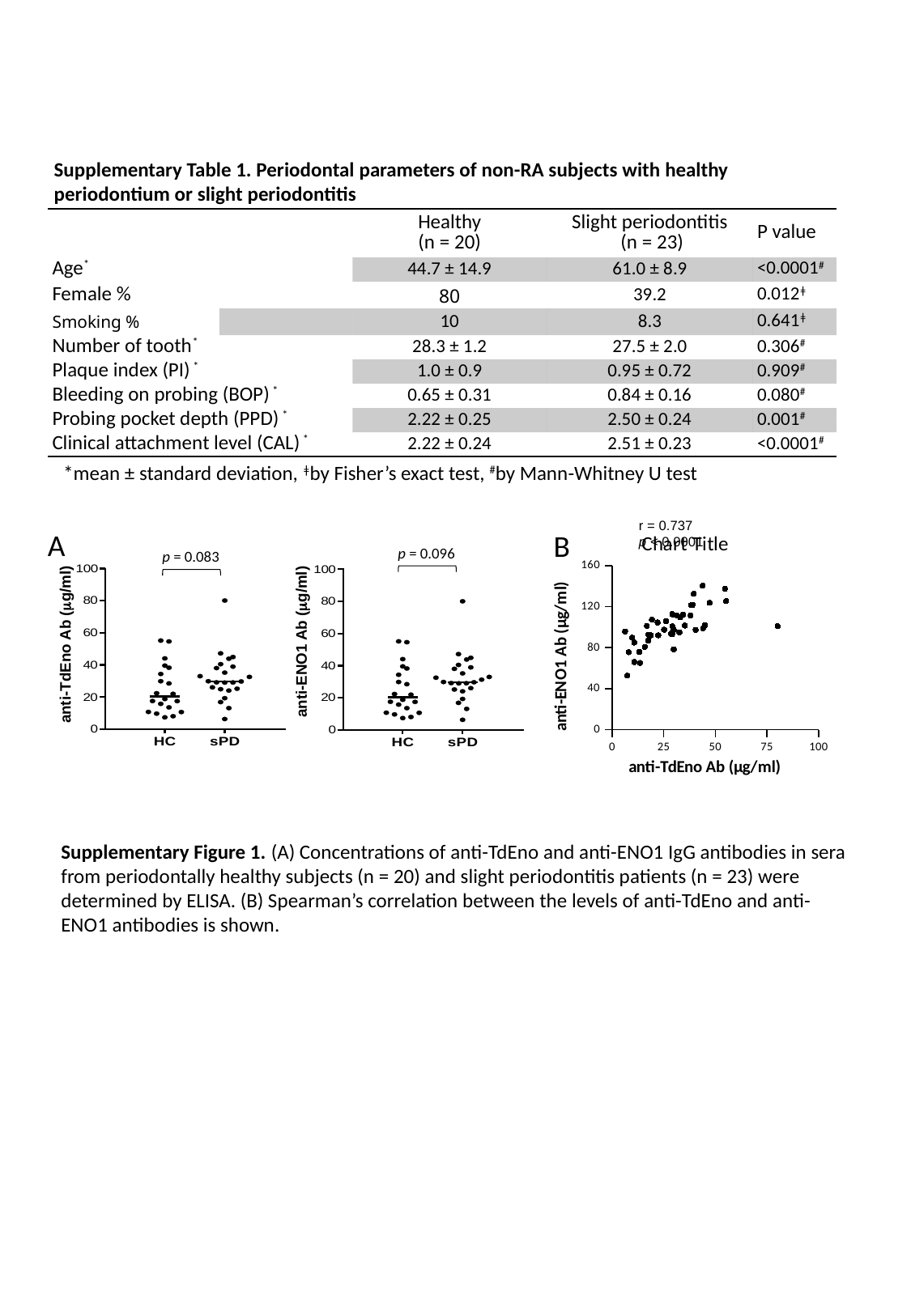

Supplementary Table 1. Periodontal parameters of non-RA subjects with healthy periodontium or slight periodontitis
| | | Healthy (n = 20) | Slight periodontitis (n = 23) | P value |
| --- | --- | --- | --- | --- |
| Age\* | | 44.7 ± 14.9 | 61.0 ± 8.9 | <0.0001# |
| Female % | | 80 | 39.2 | 0.012ǂ |
| Smoking % | | 10 | 8.3 | 0.641ǂ |
| Number of tooth\* | | 28.3 ± 1.2 | 27.5 ± 2.0 | 0.306# |
| Plaque index (PI) \* | | 1.0 ± 0.9 | 0.95 ± 0.72 | 0.909# |
| Bleeding on probing (BOP) \* | | 0.65 ± 0.31 | 0.84 ± 0.16 | 0.080# |
| Probing pocket depth (PPD) \* | | 2.22 ± 0.25 | 2.50 ± 0.24 | 0.001# |
| Clinical attachment level (CAL) \* | | 2.22 ± 0.24 | 2.51 ± 0.23 | <0.0001# |
*mean ± standard deviation, ǂby Fisher’s exact test, #by Mann-Whitney U test
r = 0.737
p < 0.0001
### Chart:
| Category | |
|---|---|A
B
p = 0.096
p = 0.083
anti-TdEno Ab (mg/ml)
anti-ENO1 Ab (mg/ml)
Supplementary Figure 1. (A) Concentrations of anti-TdEno and anti-ENO1 IgG antibodies in sera from periodontally healthy subjects (n = 20) and slight periodontitis patients (n = 23) were determined by ELISA. (B) Spearman’s correlation between the levels of anti-TdEno and anti-ENO1 antibodies is shown.

## Slide 2
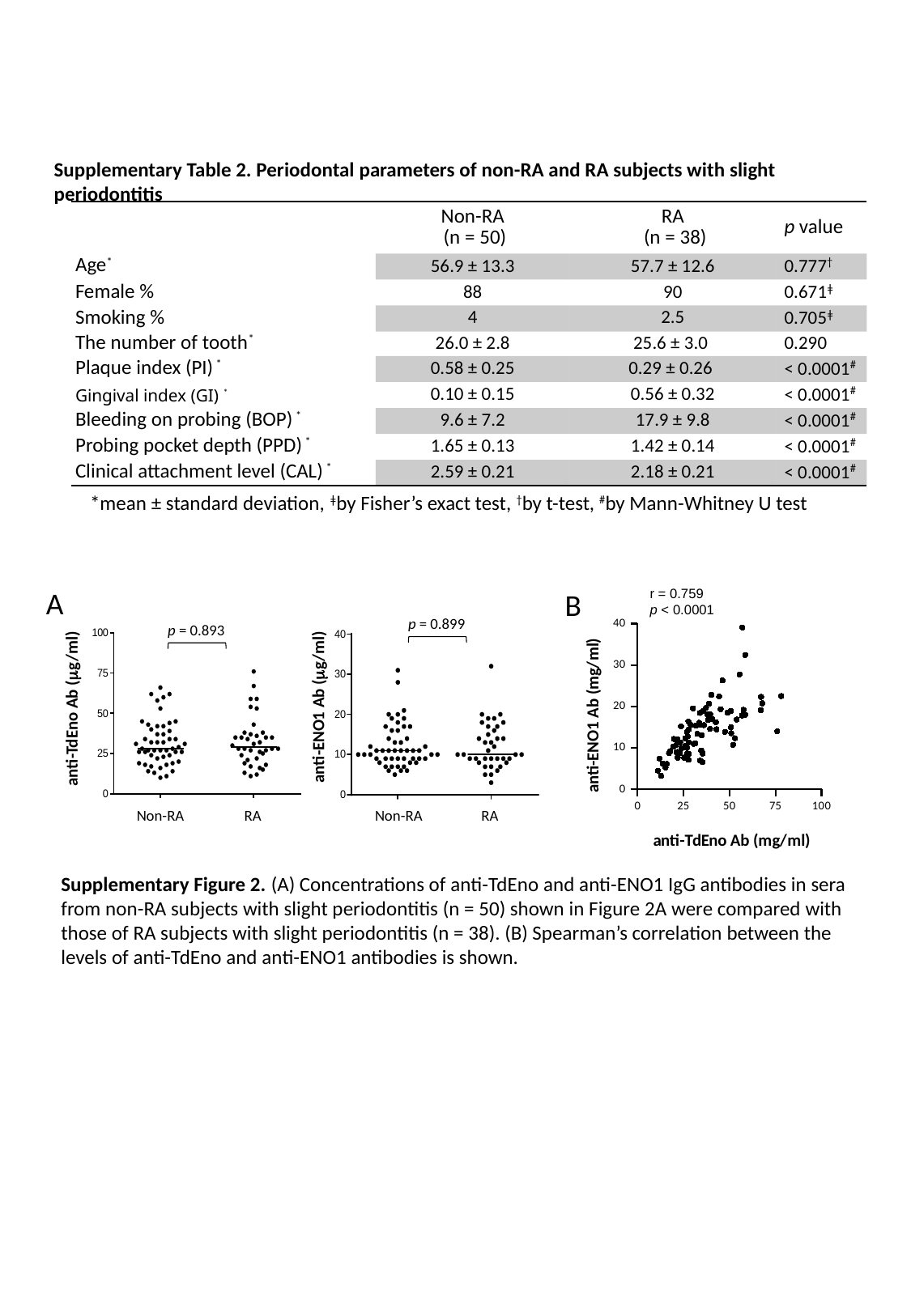

Supplementary Table 2. Periodontal parameters of non-RA and RA subjects with slight periodontitis
| | | Non-RA (n = 50) | RA (n = 38) | p value |
| --- | --- | --- | --- | --- |
| Age\* | | 56.9 ± 13.3 | 57.7 ± 12.6 | 0.777† |
| Female % | | 88 | 90 | 0.671ǂ |
| Smoking % | | 4 | 2.5 | 0.705ǂ |
| The number of tooth\* | | 26.0 ± 2.8 | 25.6 ± 3.0 | 0.290 |
| Plaque index (PI) \* | | 0.58 ± 0.25 | 0.29 ± 0.26 | < 0.0001# |
| Gingival index (GI) \* | | 0.10 ± 0.15 | 0.56 ± 0.32 | < 0.0001# |
| Bleeding on probing (BOP) \* | | 9.6 ± 7.2 | 17.9 ± 9.8 | < 0.0001# |
| Probing pocket depth (PPD) \* | | 1.65 ± 0.13 | 1.42 ± 0.14 | < 0.0001# |
| Clinical attachment level (CAL) \* | | 2.59 ± 0.21 | 2.18 ± 0.21 | < 0.0001# |
*mean ± standard deviation, ǂby Fisher’s exact test, †by t-test, #by Mann-Whitney U test
A
r = 0.759
p < 0.0001
B
p = 0.899
anti-TdEno Ab (mg/ml)
### Chart
| Category | |
|---|---|p = 0.893
anti-ENO1 Ab (mg/ml)
Non-RA
RA
Non-RA
RA
Supplementary Figure 2. (A) Concentrations of anti-TdEno and anti-ENO1 IgG antibodies in sera from non-RA subjects with slight periodontitis (n = 50) shown in Figure 2A were compared with those of RA subjects with slight periodontitis (n = 38). (B) Spearman’s correlation between the levels of anti-TdEno and anti-ENO1 antibodies is shown.
